# Taking gambles at face value: Effects of emotional expressions on risky decisions

**DOI:** 10.3389/fpsyg.2022.958918

**Published:** 2022-10-13

**Authors:** Piotr Winkielman, Jennifer L. Trujillo, Boris Bornemann, Brian Knutson, Martin P. Paulus

**Affiliations:** ^1^Department of Psychology, University of California, San Diego, San Diego, CA, United States; ^2^Faculty of Psychology, SWPS University, Warsaw, Poland; ^3^Independent Researcher, Berlin, Germany; ^4^Department of Psychology, Stanford University, Stanford, CA, United States; ^5^Laureate Institute for Brain Research, Tulsa, OK, United States

**Keywords:** emotion, faces, decisions, expressions, choice, judgment

## Abstract

Emotional facial expressions are ubiquitous and potent social stimuli that can signal favorable and unfavorable conditions. Previous research demonstrates that emotional expressions influence preference judgments, basic approach-avoidance behaviors, and reward learning. We examined whether emotional expressions can influence decisions such as choices between gambles. Based on theories of affective cue processing, we predicted greater risk taking after positive than negative expressions. This hypothesis was tested in four experiments across tasks that varied in implementation of risks, payoffs, probabilities, and temporal decision requirements. Facial expressions were presented unobtrusively and were uninformative about the choice. In all experiments, the likelihood of a risky choice was greater after exposure to positive versus neutral or negative expressions. Similar effects on risky choice occurred after presentation of different negative expressions (e.g., anger, fear, sadness, and disgust), suggesting involvement of general positive and negative affect systems. These results suggest that incidental emotional cues exert a valence-specific influence of on decisions, which could shape risk-taking behavior in social situations.

## Introduction

The question of how emotions influence decisions has a long history in psychology, going back to Darwin who conceptualized emotions as adaptations that facilitate fitness-enhancing choices. In his treatise on emotional facial expressions, Darwin suggested that negative expressions, such as fear or anger, signal unfavorable conditions whereas positive expressions, such as happiness, signal favorable conditions (Darwin, 1998). Here, we examine whether exposure to emotional facial expressions can influence people’s risky decisions. Following current theoretical models that emphasize a valenced (positive–negative) organization of early responses to affective cues, we hypothesize that participants will be more willing to take a risky gamble after brief exposure to positive expressions than exposure to negative or neutral expressions, even if these expressions are incidental and carry no predictive value for decision-making. This hypothesis has implications for our understanding of how emotion cues influence decisions.

### Effects of emotional facial expressions

Emotional facial expressions are ubiquitous and potent social stimuli (Ekman, 1984). The processing of facial expressions can occur implicitly, even when they are irrelevant to the task, such as when participants classify faces on gender (Critchley et al., 2000; Oehman et al., 2000). Implicitly processed expressions can influence a variety of affective responses, ranging from simple preference judgments to immediate approach-avoidance behaviors. For instance, after a brief presentation of a happy face, as opposed to an angry face, participants rate novel stimuli more favorably, rate congruent stimuli more quickly, show approach-related behaviors, and consume more of a novel drink (Niedenthal, 1990; Murphy and Zajonc, 1993; Winkielman et al., 1997, 2005a; Marsh et al., 2005; Seidel et al., 2010; Anderson et al., 2012; Yang and Yeh, 2018). Interestingly, the influence of facial expressions on behavior occurs regardless of whether they change subjectively-reported feelings, suggesting mediation *via* relatively early, direct mechanisms rather than inferences from conscious feelings (Winkielman et al., 1997, 2005a; Schwarz and Clore, 2003). Furthermore, the effects of implicit emotional expressions are differentiated primarily on general positive–negative valence, rather than more specific emotional qualities (Zajonc, 2000). For example, implicit exposure to different negative expressions, such as anger vs. fear, leads to similar decreases in preference and triggers similar physiological responses (Winkielman et al., 2005b).

Theoretical accounts of these findings propose that implicitly processed emotional cues, such as facial expressions, initially activate the core affect systems responsible for a general positive–negative modification of behavior (Lang, 1995; Panksepp, 1998; Cacioppo and Berntson, 1999; Zajonc, 2000; Hamm et al., 2003; Russell, 2003; Knutson and Greer, 2008; Barrett, 2017). Specifically, happy expressions that signal a favorable environment activate the positive affect system, whereas fearful or angry expressions that signal an unfavorable environment activate the negative affect system, with more differentiated reactions requiring additional processing of the expression and its situational context (an issue we return to in the discussion). These theoretical accounts also highlight that the positive affect system promotes exploration and focus on the possible rewards, whereas the negative affect system promotes caution (Cacioppo and Berntson, 1999; Knutson and Greer, 2008). These considerations suggest that implicit exposure to positive expressions should temporarily promote risk-seeking whereas exposure to negative expressions should promote risk-avoidance. We return to the specific mechanisms in the discussion.

### Emotion and risky decisions

Traditionally, decision making under risk was seen as a primarily cognitive process requiring integration of information about the probability and desirability of outcomes (Kahneman and Tversky, 1979). Over the past three decades, however, researchers began to emphasize the importance of emotion in decision (Mellers et al., 1999; Loewenstein et al., 2001; Slovic et al., 2002; Winkielman et al., 2007; Paulus and Yu, 2012).

The general role of affect in risky decisions is highlighted by effects of lesions to the ventromedial prefrontal cortex (vmPFC)—a brain region involved in emotion (Damasio, 1994). Bechara et al. (1997), using Iowa Gambling Task (IGT), showed that, compared to controls, vmPFC-lesioned patients tend to disadvantageously choose gambles that are initially attractive, but subsequently associated with big losses, presumably because the patients lack affective feedback associated with big losses. However, in some circumstances, vmPFC-lesioned patients can also choose advantageously. Shiv et al. (2005) demonstrated this using a myopic loss-aversion task involving repeated choices between a risky, but more profitable “invest” option, and a safe, but less profitable “pass” option (Gneezy and Potters, 1997). In this task, it behooved participants to always invest because the expected value on each round was higher if they invested than if they did not ($1.25 vs. $1). Interestingly, compared to controls, vmPFC patients invested more frequently (and made more money), presumably because they lack negative affective feedback associated with occasional investment losses (Shiv et al., 2005).

The possibility that emotional expressions may influence decisions is raised by reports that emotional faces bias learning of advantageous choices. Specifically, in one study participants with social anxiety performed the Iowa Gambling Task (IGT) modified so that decks of card were marked with happy or angry expressions. The results showed a bias against choosing decks marked with angry faces, especially in the early trials where the outcome contingencies if IGT are unknown (Pittig et al., 2014). In another line of research, participants learned reward probabilities associated with a happy versus an angry face and showed overweighing the positive outcomes associated with happy faces, especially early in learning (Averbeck and Duchaine, 2009). One interpretation of this line of studies is that when facial expressions are a part of the task, participate assume them to be informative about reward probability, use them to guide their initial decisions and later integrate them with more diagnostic task information. The current studies investigate the possibility that facial expressions influence decisions even when they are implicit and when the decision task does not involve learning and has a probabilistic structure, thus reflecting changes in participants’ risk-seeking or risk-aversion (Kahneman and Tversky, 1979). This prediction is grounded in the frameworks known as “risk-as-feelings” (Loewenstein et al., 2001; Slovic et al., 2002) and “anticipatory-affect” (Knutson and Greer, 2008), which we elaborate on in the discussion.

### Current research

The current studies build upon previous work by exploring whether risky decisions are influenced by implicit emotional cues, such as facial expressions. The current studies are important for several reasons. First, as discussed, prior studies showed that facial expressions influence basic preference judgments, quick approach-avoidance decision, simple approach-related behaviors, or choices of rewards in probabilistic learning tasks. However, there is a need for studies that explore the influence of these ubiquitous socio-emotional cues when are implicit, and with decision tasks do not involve learning but have a fixed probabilistic structure and thus can reveal changes in risk attitudes (Kahneman and Tversky, 1979). Second, many prior studies on emotion and risky decision explored effects of hedonically undifferentiated feedback, such as arousal, in neurological patients (e.g., Bechara et al., 1997; Shiv et al., 2005), or negative cues, such as angry faces, with participants with anxiety (e.g., Pittig et al., 2014), or fearful faces, especially for low-dominance participants (Schulreich et al., 2016). In contrast, we test a directional valence hypothesis, which predicts greater risk taking after viewing positive rather than negative facial expressions, in healthy participants. Third, prior studies explored the effects of subjective feeling states, such as general positive and negative mood, or specific emotions such happiness, anger, or fear (Johnson and Tversky, 1983; Raghunathan and Pham, 1999; Lerner and Keltner, 2001; Fessler et al., 2004). Accordingly, these studies used manipulations designed to elicit an enduring change in subjective feeling (e.g., recall of emotional memories, reading an evocative scenario, watching movies, or listening to music). Those studies also focused on how subjective feelings of the same valence, but different quality, can have distinct effects on risky decisions (e.g., feeling fearful increases risk perception whereas feeling angry decreases it, Lerner and Keltner, 2001). In contrast, the current studies focus on the effects of implicit emotional cues, such as facial expressions. Based on previously mentioned work with facial expressions, and theoretical accounts of affective cue processing, we hypothesized that implicit emotional expressions would have a general valenced effect on risk decisions—with positive expression increasing risk seeking and negative expression decreasing it. To test this hypothesis, in our tasks we implicitly presented happy, neutral, angry, disgust, sad, and fearful expressions, before participants made decisions between options that varied in risk.

### Current paradigms

To study the effect of implicit facial impressions on risk taking behavior, we used three different decision tasks. Each task involved multiple trials in which participants first saw a facial expression and then choose between options that varied in risk. Study 1 employed a Pass/Invest task based on the myopic gambling paradigm described earlier. Study 2 used the same task, but with facial expressions being presented very briefly, near detection threshold. Studies 3 and 4 employed two gambling tasks from the decision-making literature—three Cards and Risky Gains (described shortly)—to examine the impact of emotional facial expressions across different (i) payoff structures, (ii) operationalizations of risk, (iii) temporal dimensions of the decision, and (iv) types of negative facial expressions. In each study, the valence of facial expression was randomized and thus carried no information about the decision. As described in details below, we based sample sizes on previously published papers. We did not conduct *a-priori* power analyses but report post-hoc (observed) power analyses based on actual effect sizes in current studies. In each study, we excluded (if relevant) participants who gave the same response on each decision trial. Additional criteria (e.g., suspicions) are discussed in the method section of each study.

## Study 1

Study 1 used the myopic loss aversion task (Pass/Invest) to examine whether emotional cues influence risky decisions of healthy participants in a valence-specific way (Gneezy and Potters, 1997; Shiv et al., 2005). We modified the task so that prior to each investment decision, participants were shown a face. To ensure that participants saw each face, and to make its emotion unobtrusive, they were asked to indicate the face’s gender (male/female). To manipulate valence, the faces varied in emotional expression (angry, neutral, or happy). Because participants’ goal was to maximize their account, the optimal, profit maximizing strategy in this task is to invest on every round. However, we predicted that participants would fail to invest on some portion of the trials, replicating previous studies on myopic loss aversion. More importantly, we also predicted that the likelihood of investing on each trial would be influenced by the valence of the emotional expressions, with angry faces decreasing investing relative to happy faces.

We also examined whether the influence of emotional cues on gambling decisions occurred only when participants were informed about the immediate outcome of each gamble, or also when participants were informed about their cumulative wealth. This is interesting because the cumulative information might promote a more long-term strategy (Gneezy and Potters, 1997). Accordingly, on each trial, some participants received information only about the outcome of the current trial, whereas others also received information about their cumulative earnings. An effect of emotional cues on decisions in both conditions would suggest that their influence extends beyond situations involving consideration of only single gambles.

### Method

#### Participants and procedure

Forty-seven undergraduates participated for course credit. Three participants (6%) were excluded from the analyses because they made the same response (invest) on each gambling trial (this did not change any effects). Gender and age information was not collected but the participant population is about 75% female and has a mean age of about 20.

**Figure 1:**
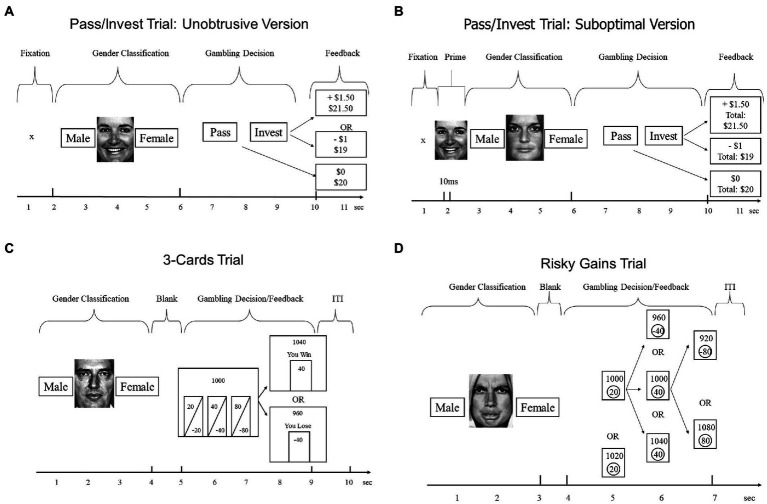
Trial structures of tasks used in our four experiments. Study 1 (Panel **A**), Study 2 (Panel **B**), Study 3 and 4 (Panel **C**,**D**), and Study 4 (Panel **C**). The specific numbers in feedback events represent possible outcomes on the first trial.

Participants were tested individually using a computerized version of the myopic loss aversion paradigm (Figure 1, top panel). Participants were told that the experiment involved multiple trials of two separate but interleaved tasks: (i) a gender classification task, in which the goal was to perform as accurately as possible, and (ii) a gambling task, in which the goal was to earn as much play money as possible. The trial started with a fixation star. In the gender classification part, a face appeared for the total of 4 s. After the first 2 s of face presentation, participants were asked a question about its gender (male vs. female) and had 2 s to answer it. Faces came from the classic set by Ekman and Friesen (1975), varied in emotional expression (angry, neutral, or happy), and were cropped to remove obvious gender cues such as hair.

Each gender-classification trial was immediately followed by a gambling trial designed after Shiv et al. (2005). Beginning with an account of $20, participants decided on each round whether to invest $1 from their account for a 50% chance of winning an additional $1.50 vs. losing $1, or whether to pass on that round and keep $1 in the account. Note that the expected value (EV) for investing equals $1.25, whereas EV for passing equals $1, making investing the profit-maximizing choice. After each gambling decision, the computer displayed feedback with the trial outcome ($0 if pass, $1.50 if win, −$1 if loss). In addition, for some participants, the computer also displayed the cumulative total (e.g., $ 21.50 if the participant won on the first trial), before advancing to the next of 54 total trials. As shown in Figure 1, participants had only 4 s to make the decision between the pass and invest options, which decreased the opportunity for deliberation.

#### Power analysis

We did not conduct an *apriori* power analysis. Our decision about the sample size was made based on the original sample size in Shiv et al. (2005), which had 19 healthy participants. However, we performed *post-hoc* (observed) power analyses using three different methods. One was implemented in the R package Superpower. For the simulation, we assumed our analysis design and the observed empirical distribution of our data. Results show that the observed power to detect the effect of Face Valence category was 92%, [CI 90.15, 93.53]. Interestingly, as described below, using our experimental data and design, the SPSS 28 option Observed Power in repeated measures ANOVA, yielded a lower, but still respectable estimate of 0.80 for the Face Valence effect (η^2^ = 0.11). This estimate was close to analyses using G-Power (Faul et al., 2007) which calculated 78% observed power for the Face Valence effect (assuming effect size 0.11, *N* = 44, 3 within subject conditions, and 18 measurements per conditions). Reflecting the convergence between the last two methods, we relied on SPSS 28 and G-Power in providing estimates of observed power in this and the remaining studies.

### Results

#### Preliminary analyses

Participants classified gender accurately (90%) and quickly (884 ms), with no differences among valence conditions or feedback conditions (*F*_s_ < 1). Analyses of the gambling decisions replicated previous findings, showing that participants invested on most trials (74%).

#### Main analyses

**Figure 2:**
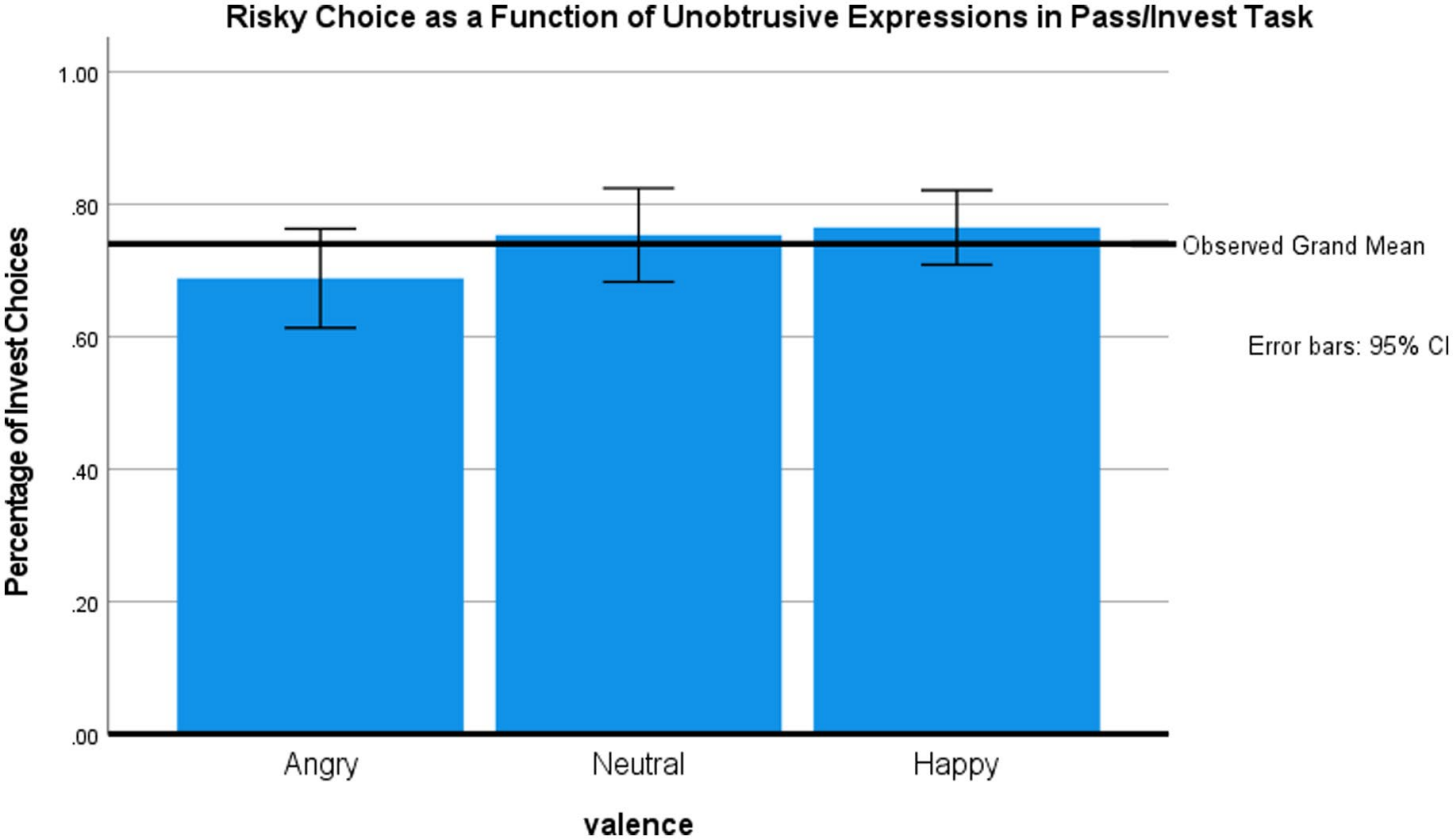
Effects of facial expression on risky choice in the Pass/Invest Task (Study 1).

To test our main hypothesis, we calculated the probability of investing as a function of the preceding facial expression (happy, neutral, and angry) and the type of trial feedback (current trial only vs. current trial and total account). Figure 2 shows the mean choice of the risky option as a function of the facial stimulus. An mixed ANOVA with face valence (within subject) and feedback (between subjects) showed the expected main effect for face valence, *F* (2, 84) = 4.93, *p* < 0.01, *η*^2^ = 0.11, observed power 80%. Simple tests (two-tailed for all tasks, no correction for multiple comparisons) revealed that participants invested less after exposure to angry versus neutral faces, *t*(43) = 2.22, *p* < 0.05, and happy faces *t*(43) = 2.4, *p* < 0.05. There were no main effects or interactions involving the feedback condition (all *F_s_* < 1), though our study was underpowered to find these secondary effects.

### Discussion

Study 1 confirmed our prediction that emotional facial expressions influence gambling decisions. Viewing an angry face decreased the likelihood of investing on the subsequent trial, as compared to neutral and happy faces. This decrease is remarkable given that investing was beneficial from the profit-maximizing perspective. The lack of differences between happy and neutral faces is consistent with proposals that decisions in the myopic loss aversion task are primarily controlled by negative affect (Kahneman and Tversky, 1979; Shiv et al., 2005)—but see Study 2 for a different pattern. The idea that participants approach the task with a “myopic” perspective, and thus become susceptible to momentary affective reactions, also fits well with our finding that emotional expressions influenced participants’ subsequent decisions regardless of whether they received feedback about only the trial outcome or also their cumulative total.

## Study 2

We hypothesized that the emotional faces influence participants’ decisions implicitly, covertly biasing them toward more or less risk taking. Alternatively, one might argue that participants strategically base their decisions on the valence of the face in the gender discrimination task. For example, participants could assume some contingency between the valence of the face and their chances to win the gamble, or perhaps respond to some demand characteristics. To test these alternative explanations we repeated the experiment, using faces presented very briefly (near detection threshold). Such implicit emotional faces have been found to influence evaluation of subsequent stimuli and consumption behavior (Niedenthal, 1990; Murphy and Zajonc, 1993; Winkielman et al., 2005a; Anderson et al., 2012; Winkielman and Gogolushko, 2018; Yang and Yeh, 2018). Though there is a debate to what extent these paradigms fully eliminate awareness of the emotional prime (Kleckner et al., 2018), their presentation parameters (milliseconds timing for the affective prime, masked by a visible neutral face) reduce opportunity for a strategic incorporation of prime valence information into later decisions. Importantly, the effect of briefly presented expressions on financial risk-taking behavior is relatively unknown. If implicitly presented emotional stimuli influenced risk-taking behavior similarly as the optimally presented stimuli in Study 1, this would challenge any explanation of the effect by deliberative strategies or demand characteristics.

The second issue investigated in this study concerns a possibility that briefly presented facial expressions solely elicit cold, “evaluative” reactions, rather than any underlying affect. We addressed this issue by measuring facial muscle activity associated with negative and positive affect using facial electromyography, fEMG (see Cacioppo et al., 1986). If the stimuli are affectively processed, they should elicit a similar pattern as reported in earlier studies on facial responding to emotional expressions, with highest corrugator activity after angry, then neutral, then happy faces (e.g., Dimberg et al., 2000; Heller et al., 2011). Finally, we tested the detectability of such stimuli in a Forced-choice task, using different kinds of masks (as described below).

### Participants and procedure

Fifty-eight psychology undergraduates (mean age = 19.8 years, 75% female) participated for course credit. This sample size was based on the Study 1. We did not conduct an *apriori* power analysis, but, as discussed earlier, this sample size had about 80% power to detect the face valence effect with size of 0.11 which we observed in Study 1. However, as we describe shortly, in Study 2, the actual effect size for Face Valence was lower (*η^2^* = 0.06), which gives the estimate of observed power at 58%. We return to this issue later.

The procedure of the task was the same as in Study 1, except for the valence of the face in the gender discrimination task, which was always neutral. Additionally, a face was presented for 10 ms immediately before the neutral face, using 17-in Viewsonic P75f + CRTMonitor with 100 Hz refresh rate. This briefly presented face was either happy, angry, or neutral. Feedback including the cumulative total money was given after each trial. Again, participants completed 54 trials.

### Measures of subjective awareness and suspicions

After the gambling part of experiment, a questionnaire was administered. This questionnaire asked if participants had noticed anything unusual about the experiment. Further, it asked directly about the subjective awareness of briefly presented primes—whether the participants had noticed briefly appearing faces.

### Forced-choice recognition task

In a later, separate part of this research, participants also performed a forced-choice-recognition task where they needed to determine the valence of briefly presented the faces. This task was a part of a different project, which measured awareness of different sets of emotional faces, presented at different durations, with different masks, and detected with different recognition strategies. The full results of the project have been published already (Bornemann et al., 2012). However, because a small section of these data are informative here, we will discuss them (none of the analyses here were previously published). Specifically, we will discuss only the forced-choice trials with the same face set (Ekman and Friesen), same presentation time (10 ms), and masking parameters (neutral face) as in the gambling task. On each trial, participants were asked to detect the valence of the briefly presented face. Some participants were encouraged to use a: (i) visual focus strategy, (ii) feeling strategy, or (iii) neutral strategy (this made no difference, so this factor will be ignored). There were a total of 12 trials, with two types of trials: (i) happy vs. neutral face, and (ii) angry vs. neutral face, using the same six happy, six angry, and 12 neutral faces as presented in the gambling section.

### Physiological measurement

Finally, we measured physiological responses during the gambling task and forced-choice task using facial electromyography and skin conductance. To measure facial activity, electrodes were placed on the left corrugator supercilii and zygomaticus major according to the guidelines of Fridlund and Cacioppo (1986), and muscle activity was recorded using a BIOPAC MP150WSW Data Acquisition System (Biopac Systems Inc., California, United States). The EMG signal was rectified and cleaned from outliers by replacing values deviating more than two standard deviations (sd) from the mean by m+/− 2SD, respectively. Data were baseline corrected, by subtracting corrugator activity in the 500 ms before face onset. As physiological signals tend to become more pronounced with time (e.g., Dimberg et al., 2000; Bornemann et al., 2012), we analyzed average activity in the last 500 ms of the 2 s mask presentation time window. Due to technical problems, data of one subject were lost. We also measured skin conductance, but these data were very noisy, presumably because the task design required multiple movements and responses (response to the prime, response to the gamble, and moving on to next trial). Also, because of the relatively fast nature of the task, and the slow nature of skin-conductance responses, it is difficult to properly interpret these data. Therefore, they will not be discussed further.

### Results

#### Preliminary analyses of the gambling behavior

Six participants were excluded from the analyses because they made the same response on every trial (this did not change any results). One participant reported in the post-questionnaire that she had noticed the briefly appearing faces (she was among the six removed participants). One participant was an outlier and had less than 50% correct answers on the gender discrimination task, and thus was removed. For other participants, accuracy in the gender discrimination task was 94.1%. Participants invested in 68% of the trials. Mean response time was 489 ms.

#### Main analyses

**Figure 3:**
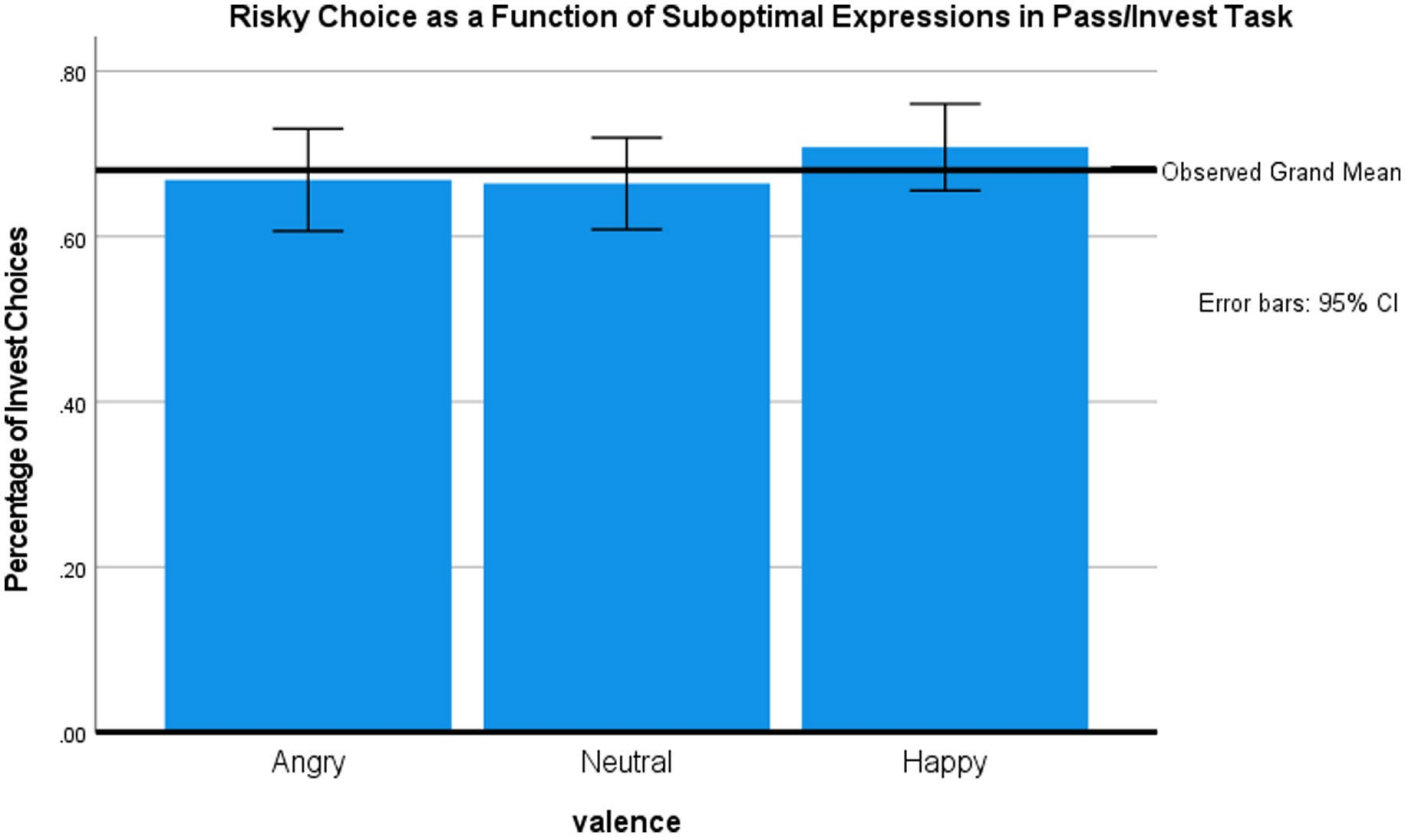
Effects of facial expression on risky choice in the Pass/Invest Task (Study 2).

We first present the analysis of choice behavior during the gambling phase. Figure 3 shows the mean percentage of risky choices by valence of the briefly presented face. A repeated measures ANOVA showed that face valence marginally influenced gambling frequency, *F* (2, 100) = 3.05, *p* = 0.052, *η*^2^ = 0.06. *Post-hoc*, two-tailed paired *t*-tests (no correction for multiple comparisons) revealed that participants gambled more frequently after exposure to happy faces (70.8%) vs. neutral faces [66.4%, *t*(50) = −2.51, *p* = 0.02] or, marginally, after happy vs. angry faces [66.9%; *t*(50) = −1.87, *p =* 0.07]. There was no difference between the influences of neutral vs. angry faces.

**Figure 4:**
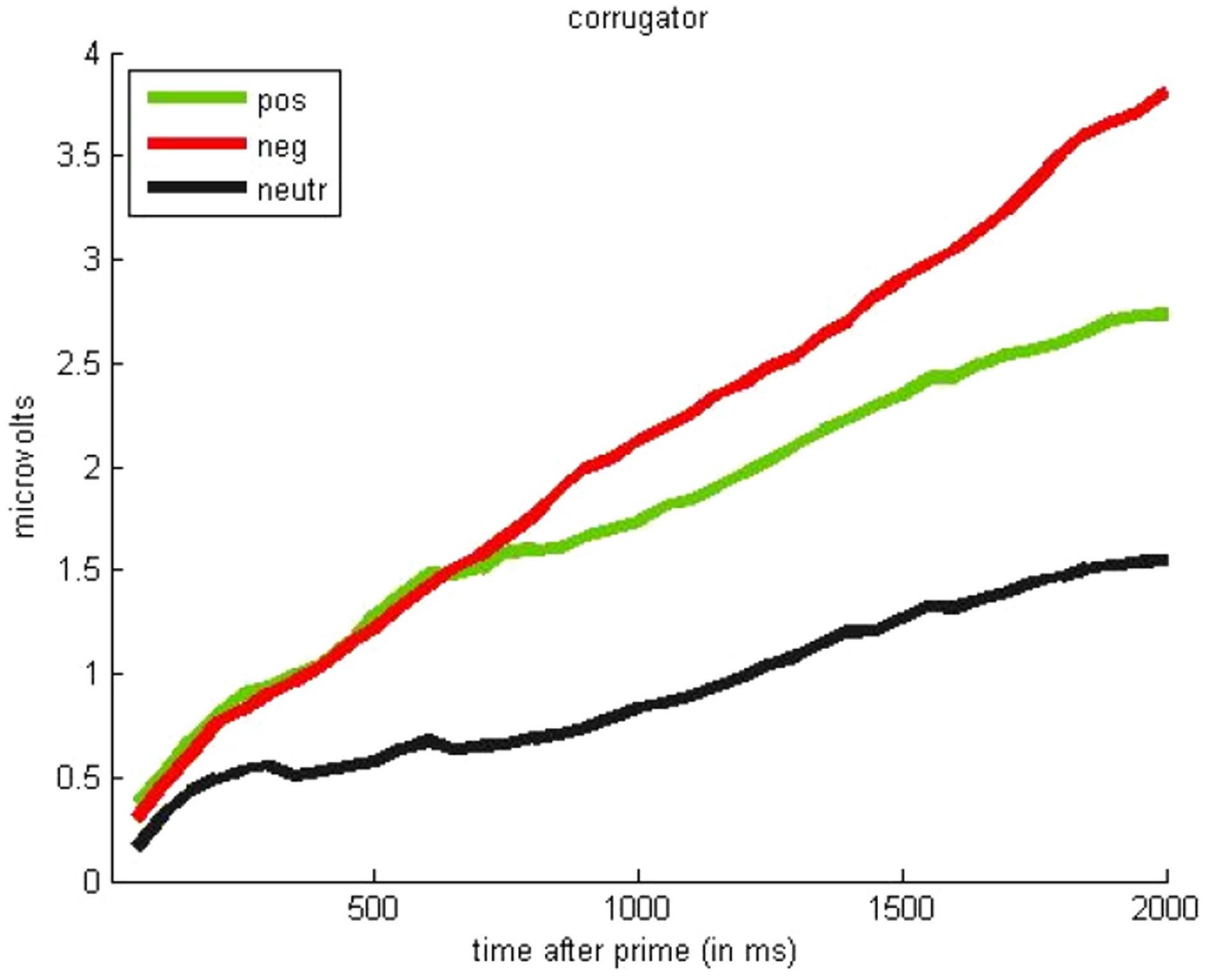
Effects of facial expression on physiological response of the corrugator supercilii in the gambling task (during the 2,000 ms after face presentation; Study 2).

Next, we analyzed facial reactions during the gambling task. We found no effect of prime valence on reactions of the zygomaticus muscle (all *F*_s_ < 1). However, as shown in Figure 4, the corrugator muscle showed some sensitivity to valence, and mostly differed as a function of face affectivity [valenced vs. neutral, *F*(2,48) = 6.07, *p* < 0.01]. Specifically, corrugator activity was stronger for angry faces vs. neutral faces, *t*(49) = 3.13, *p* < 0.01, but also stronger for happy vs. neutral faces, *t*(49) = 3.16, *p* < 0.01. We expected more robust valence differentiation because corrugator typically activates more to negative than positive stimuli. However, corrugator also responds to effort in choice tasks (Van Boxtel and Jessurun, 1993). Thus, in the context of the gambling task (with participants deliberating about a decision), this may provide post-hoc evidence for increased engagement in decisions preceded by valenced primes—though decision response times do not differ significantly as a function of different prime valences, *F*(2,49) = 0.26, *p* = 0.77.

#### Subjective awareness questionnaire and forced-choice task

As mentioned, on the subjective awareness survey, only one subject reported seeing briefly presented faces during the gambling task (and the person was removed from all analyses). Interestingly, in the forced choice task, designed to measure objective awareness, participants detected the correct face on 58% of the trials, which significantly exceeded 50% chance performance, *t*(50) = 2.88, *p* < 0.01. This suggests that when participants explicitly focus their attention on detecting the valence of the primes, it is possible to perform above chance. Consequently, it is more appropriate to describe the brief, 10 ms primes in the current experiment as unattended, implicit, or suboptimal rather than subliminal or unconscious (see also Kleckner et al., 2018). Also, let us note two findings from the same participants that were reported as a part of different project, using a larger set of emotional faces, presentation durations, and different detection strategies (see Bornemann et al., 2012). First, facial EMG responses during a task testing valence detection from faces differentiated valence of briefly presented expressions (10 and 20 ms), as reflected in significantly greater corrugator responses to brief angry, rather than happy facial expressions. Second, participants detected happy expressions more easily than angry expressions, as reflected in significantly higher discrimination performance (see Bornemann et al., 2012).

### Discussion

The results of Study 2 indicate that briefly presented emotional faces influence gambling decisions. Unlike Study 1, happy but not angry faces influenced subsequent decisions as compared to neutral faces. This stronger influence of briefly presented happy faces has been reported in other paradigms that involve subsequent evaluations of neutral stimuli (Phaf et al., 2005; Dannlowski et al., 2007). These findings, and our observed difference between unobtrusive influence (Study 1) vs. suboptimal influence (Study 2), may reflect the fact that smiling, as opposed to angry, faces are easier to detect under brief exposure conditions because they contain more low spatial frequencies or have a more distinct facial configuration (Phaf et al., 2005; Smith and Schyns, 2009). In fact, the results from the forced-choice test are consistent with this conclusion (Bornemann et al., 2012).

This study suggests that emotional faces can influence gambling behavior even when they are barely noticed by the participants, as evidenced by low detection rates in forced-choice task. This reduces the potential concern that facial effects on gambling are due to participants’ conscious strategies or demand characteristics. Unsurprisingly, the comparison of effect sizes suggests that influence of suboptimally (10 ms) presented faces (Study 2, η^2^ = 0.06) is weaker than influence of unobtrusively (4 s) presented faces (Study 1, η^2^ = 0.11). Still, future studies might directly compare both presentation conditions (Murphy and Zajonc, 1993), including using physiological measures (Lapate et al., 2014).

Facial muscular recordings (EMG) suggest that the emotional faces are processed, at least partially, and can elicit bodily reactions. These changes in participants’ own bodily responses suggest that the effects of the faces on gambling choices observed in Studies 1 and 2 involve affective processing, rather than purely cognitive mechanisms. We return to this issue in General Discussion.

## Study 3

In Study 3, we addressed several conceptual issues using two different gambling tasks from the decision literature—the “3-Cards Task” and “Risky Gains Task,” explained in detail shortly (Paulus et al., 2003; Leland and Paulus, 2005).

First, we examined whether positive and negative expressions had a similar, valence-specific impact on risk decisions, even when risky choice does not maximize returns. Note that, in Studies 1 and 2, the risky option (Invest) had a higher expected value than the safe option (Pass). This raises the possibility that positive expressions promote risk seeking only when a risky option is inherently beneficial. This could be due to evaluative matching, which promotes the choice of options whose value matches the evaluative context (Musch and Klauer, 2003), or because positive affect promotes reliance on prepotent or dominant tendencies, preferred responses, or default inclinations (Clore et al., 2018). To rule out these possibilities, the tasks in Study 3 included options with equal expected values.

Second, we examined whether facial expressions influence choices only between safe and risky options, or also among options that are all uncertain but vary in degrees of risk (see Anderson, 2003, for discussion). Therefore, in the 3-Cards task, we manipulated risk by keeping the probability of each option the same, and varying the variance of outcomes, with the largest variance option being the riskiest by definition (Dickhaut et al., 2003). We expected that positive expressions would increase the probability of choosing the riskiest option.

Third, we examined whether emotional expressions would have similar effects even when the risky option is not the initial, “default” choice. It is important to test this possibility because affective cues could influence both impulsive behavior (Gray, 1990; Clore et al., 2018) and amount of cognitive processing (Mackie and Worth, 1991). For example, in Studies 1 and 2, more positive (or more negative) expressions may have changed participants’ decision about accepting (or rejecting) the initial, or “default” option. To address this possibility, the Risky Gains task in Study 3 presented options in a sequential, rather than simultaneous fashion, with the safe option offered first, followed by risky options.

Fourth, we examined whether the influence of facial expressions depended upon general valence (positive vs. negative) or the specific emotional quality of the expression. Studies 1 and 2 raise the possibility that changes in risky choice after viewing an emotional face could be uniquely due to specific emotion (e.g., anger), rather than to the general valence of the expression. To investigate this issue, we included an additional negative expression (fear) in both tasks of Study 3. We predicted that both fear and anger would have similar effects, consistent with the idea that implicit facial expressions activate general positive and negative affect systems (Cacioppo and Berntson, 1999; Barrett, 2017).

Finally, we addressed two auxiliary issues. To examine whether the effects generalize across different presentations of probabilities, in the 3-Cards task we informed participants about the exact likelihood of a gain and a loss for each option, whereas in the Risky Gains task we left this information unspecified. Furthermore, to examine the possible role of subjective experience, in both tasks we asked participants to report whether the faces influenced their feelings. As discussed, based on previous research, we expected similar effects for participants whether they reported changes in feelings or not (Winkielman et al., 2005a).

### Method

#### Participants and procedure

Twenty-four undergraduates participated for course credit. One participant was excluded for always choosing the same option (high card). Participants performed both the 3-Cards task and the Risky Gains task, with task order counterbalanced. The sample size was based on previous research using this task, which tended to have samples around 20 participants (Paulus et al., 2003; Leland and Paulus, 2005). We did not conduct an *apriori* power analysis for this study, but we did conduct a *post-hoc*, observed power analysis for both tasks in G-Power and in SPSS 28. In the Risky Gains task, G-power suggests we had 94% power to detect the observed effect size of = 0.17 (four within-subject levels, 24 trials per level). SPSS estimates of power for this task give observed effect size is around 87%. For the 3-cards task, G-power suggests we had 53% power to detect the observed effect size of 0.12 (four within-subject levels, 14 observations per level). SPSS estimates, below, are higher (around 70%). Still, overall this means that 3-card task in Study 3 was underpowered, but see Study 4 for an extension with the same task.

The structure of both tasks was similar to task structure in Study 1, with multiple trials of interleaved gender classifications and gambling decisions. For both tasks, participants were instructed to classify gender as accurately as possible, and to earn as many points as possible on the gambling portion, starting with an initial endowment of 1,000 points. The gender classification portion for both tasks was similar to classification in Study 1, except that fearful faces were presented in addition to angry, neutral, and happy faces. Further, the duration of face presentation was adjusted to match the length of the respective gambling trial (4 s in the 3-Cards, 3 s in Risky Gains). The gambling portion of each trial immediately followed the gender decision and differed between the two tasks as described below.

#### The 3-cards task

Participants chose from three simultaneously presented options (Figure 1, left bottom panel). Participants were informed that all options had equal probabilities (50/50%) but varied in how much could be gained or lost. The options appeared for 4 s as gambles of 20/−20, 40/−40, and 80/−80 points. After participants made their choice, the computer displayed the outcome of the gamble and updated the total account, before advancing to the next of 56 total trials.

#### The risky gains task

Participants chose from three sequentially presented options (Figure 1, bottom right panel). Participants were told that the 20-point option was a sure win, whereas the 40 and 80 options could win or lose 40 or 80 points, respectively. The options appeared in ascending order (20-40-80) for 1 s each. Unlike the other tasks, participants were not told the probabilities of the two risky options. The actual probabilities were such that each participant’s final score was identical whether he or she selected the 20, 40, or 80 option. At the end of each trial, the computer displayed the outcome of the gamble and updated the total, before advancing to the next of 96 total trials (see Paulus et al., 2003).

### Results

#### Preliminary analyses

Because there were no main effects or interactions involving task order (*F_s_* < 1.2), subsequent analyses collapsed across this variable. In both tasks, gender classification was accurate (94% in 3-Cards and 93% in Risky Gains), relatively fast (1,650 ms in 3-Cards and 1,529 ms in Risky Gains) and did not vary by expression (*F*_s_ < 1.4).

A post-experimental questionnaire given after each task asked: “Did the faces you were presented with have any influence on your feelings?” The “yes” or “no” response (48% yes in both tasks) did not moderate the impact of expressions (both *F*_s_ < 1). The questionnaire also asked “What strategy and information did you use to gain the most points?” Seventy-nine percent of participants mentioned strategies unrelated to expressions. For both tasks, the response to this question did not moderate the impact of expressions (*F*_s_ < 1).

#### 3-cards task

**Figure 5:**
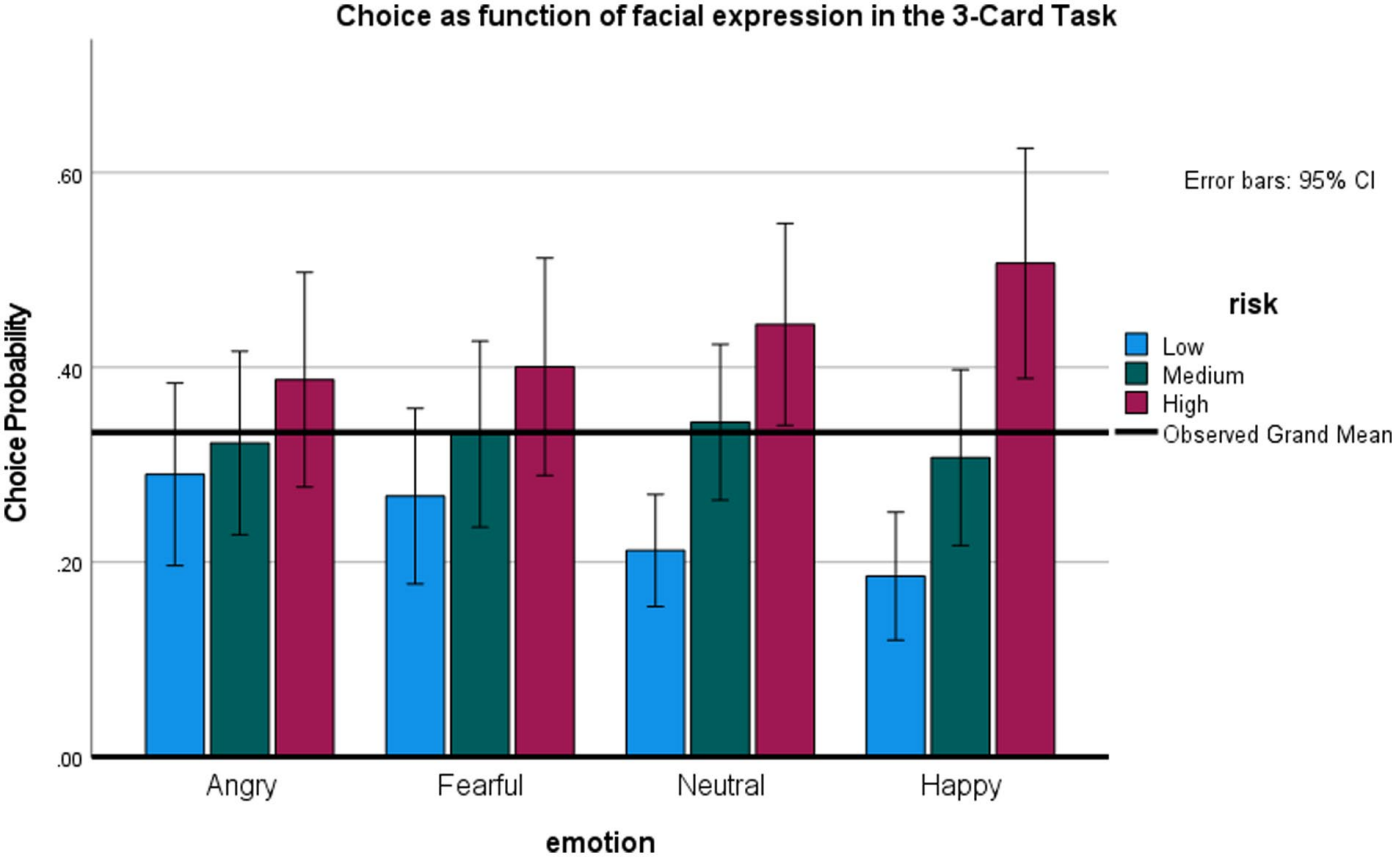
Effects of facial expression on risky choice in the 3-Card Task (Study 3).

This task examined whether facial emotions influence choice when all options are risky. Figure 5 presents the percentage of low, medium, and high-risk cards selected as a function of preceding expression. Note that for each emotion level, the individual card choices are mutually exclusive at the single trial level, and overall must all add to 100% (because the choice of more high-risk cards necessitates that fewer low-risk and medium risk-cards are chosen). Note also that our analytic choice was to analyze the effects of emotion on the low-risk and high-risk options separately—even though they are not fully independent—but allowed us to use the repeated measures ANOVA framework, as in previous studies.

An repeated measures ANOVA on the probability of choosing the riskiest option (+/−80) showed an overall effect for expression, *F* (3,66) = 3.17, *p* < 0.05, *η^2^* = 0.13, observed power 71%. Participants were most likely to select the riskiest option after happy, relative to angry, *t* (22) = 2.67, *p* < 0.05, or, marginally, fearful expressions, *t* (22) = 1.95, *p* = 0.06, all tests two-tailed, no correction for multiple comparisons. An ANOVA on the least risky option (+/− 20) also showed an overall effect of expression, *F* (3, 66) = 2.71, *p* = 0.05, *η^2^* = 0.11, observed power 63%. The probability of selecting the safest option was smallest after an angry, *t* (22) = 2.2, *p* < 0.05 and, marginally, fearful, *t* (22) = 1.92, *p* < 0.07, relative to a happy expression. There were no differences between effect of anger versus fear, and effects of expression on the medium-risk option (+/− 40).

#### Risky gains task

**Figure 6:**
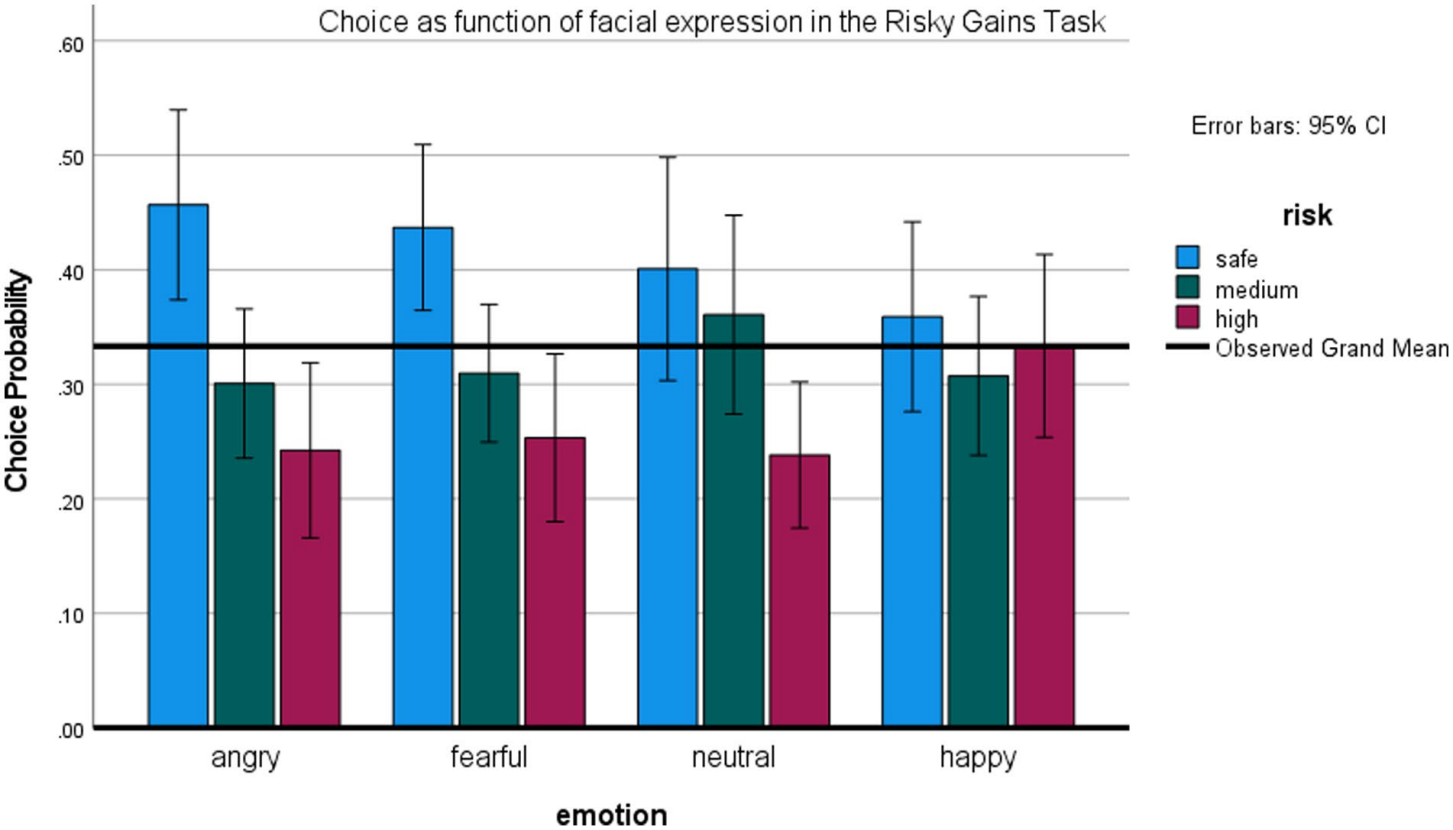
Effects of facial expression on risky choice in the Risky Gains Task (Study 3).

This task examined whether facial expressions influence choice of a risky option even if one must first bypass a safe option. Figure 6 depicts the probability of selecting the riskiest (80/−80) option, medium (40/−40) and the safe (20) option as a function of the preceding expression. Again, we analyzed the effects of emotion on the low-risk and high-risk options separately, though they are not fully independent, using the repeated measures ANOVAs.

ANOVA on the riskiest option revealed a main effect of expression, *F* (3,66) = 4.58, *p* < 0.01, *η*^2 =^ 0.17, observed power 87%. Participants’ probability of selecting the riskiest option was higher after happy versus other expressions, all *t*s (22) > 2.6, all *p*s < 0.05. ANOVA on the safe option also revealed a main effect of expression, *F* (3,66) = 3.04, *p* < 0.05, *η*^2^ = 0.12. Participants were more likely to choose the safe option after an angry, *t* (22) = 2.71, *p* < 0.05, or fearful expression, *t* (22) = 3.03, *p* < 0.01, relative to happy expression. These post-hoc tests were not corrected for multiple comparisons. Again, there were no significant differences between effects of exposure to anger vs. fear expressions, and no effects of expression on the medium-risk option.

## Study 4

The preceding studies found that unobtrusively presented facial expressions influence choices in a valence-specific way, such that positive expressions (happy) tend to increase risk taking whereas negative (anger and fear) expressions reduce it. The aim of this study was to examine the effects of two other negative facial expressions—disgust and sadness. Again, consistent with the idea that implicit facial expressions activate general positive and negative affect systems (Cacioppo and Berntson, 1999; Barrett, 2017), we expected that disgust and sadness would have a similar, risk-reducing effect as compared to happy faces. However, it is also worth noting that in the classic Ekman and Friesen face set that we used, sadness is often confused with neutral faces (Arce et al., 2009). It is also an expression that in some paradigms produces approach responses, perhaps reflecting empathic concern (Seidel et al., 2010). Finally, it is an associated with low-arousal, and thus should have limited potency as a driver of risk behavior in the anticipatory affect model (Knutson and Greer, 2008).

In this study we again used the 3-card task as it has the advantage of offering participants a three alternative forced choice, which controls for potential non-specific effects of emotion on unrelated processes (e.g., impulsivity). Finally, we administered a questionnaire that asked participants for their suspicions, their interpretation of the task, and whether they have experienced any feelings in response to the facial stimuli.

### Participants and procedure

Thirty participants were run and three were excluded because they correctly guessed the hypothesis (including these participants did not change the results, see below). The procedure was identical except that instead of angry and fearful faces, we used sad and disgusted faces. At the end of the task, we also interviewed participants extensively about any possible suspicions regarding the link between the faces and gambles. Reaction time for gender identification was 1,790 ms and did not vary by the type of expression. G-power indicated 50% power to detect the observed effect size of 0.10 in the 3-card task (four levels, 14 trials per level). As mentioned shortly, SPSS 28 estimate for observed power based on actual data was higher at 68%. Still, this means that Study 4 was underpowered (we later offer a joint power analysis for the 3-cards tasks in both Study 3 and Study 4).

### Results

**Figure 7:**
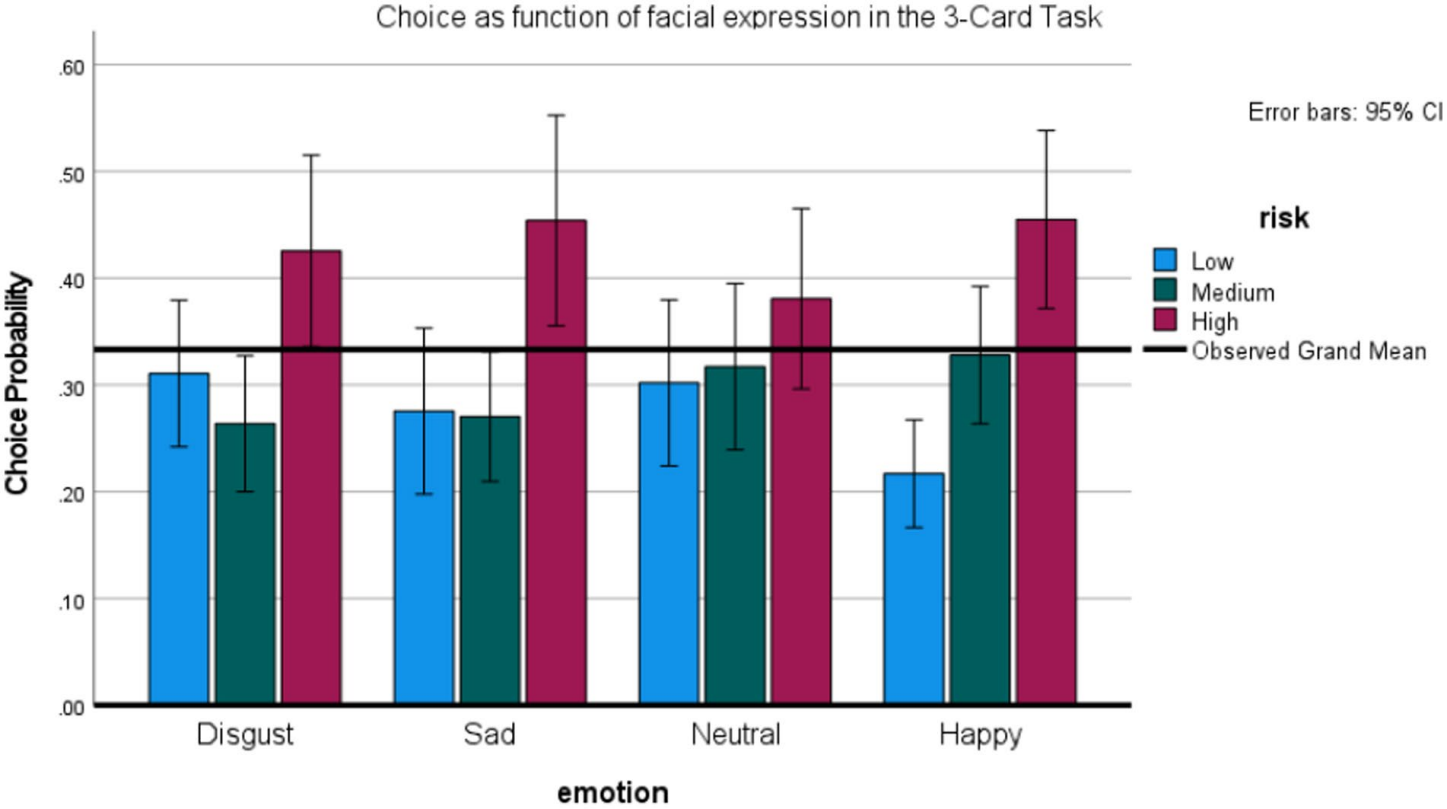
Effects of facial expression (including disgust and sadness) on risky choice in the 3-Card Task (Study 4).

Figure 7 graphs the probability of selecting the low, medium, and high-risk option as a function of preceding expression. No effects for expressions were found using repeated measures ANOVAs on the probability of choosing the riskiest option (+/−80), as well as the medium-risk option (+/− 40). However, a repeated measures ANOVA on the least risky option (+/− 20) showed a main effect of expression, *F* (3,78) = 2.98, *p* < 0.05, *η2* = 0.10, observed power = 68%. The probability of selecting the least risky option was highest after a disgust, *t* (25) = 2.7, *p* < 0.05, marginally, sad, *t* (25) = 1.84, *p* < 0.08, and neutral, *t* (25) = 2.17, *p* <. 05, relative to a happy expression (all tests two-tailed).

As mentioned, after the gambling task, we also asked participants whether they experienced any subjective feelings in response to the unobtrusive facial expressions. Only 31% of the participants said “yes.” Interestingly, there were no significant interactions between reported feelings and emotional influence (*p* > 0.30), suggesting that the observed effects were not mediated by explicitly noticed changes in fleeting affective states.

## General discussion

Together, these studies suggest that the valence of implicitly presented emotional facial expressions influences risky decisions even if the valence of the facial expression is uninformative about the decision outcome. Specifically, positive expressions (happiness) tend to increase the choice of a risky option, whereas negative expressions (anger, fear, sadness, and disgust) tend to decrease it. This effect is not specific to a particular task and occurs with different types of payoff structures, risk operationalizations, time demands, unobtrusive vs. suboptimal face presentations, and explicitly stated vs. unspecified outcome probabilities. In the Invest/Pass task of Studies 1 and 2, the risky option had a higher expected value than the safe option, but in the 3-Cards tasks of Studies 3 and 4, all risky options had equal expected value. In the Invest/Pass and the Risky Gain tasks, participants chose between safe and risky options, whereas in the 3-Cards task, all options were uncertain, with more risky options having greater variance of outcomes. In the Invest/Pass and 3-Cards tasks, participants chose among options presented simultaneously, but in the Risky Gains task, participants chose sequentially, needing to bypass a safe option to select a risky option. Finally, in the Invest/Pass and the 3-Cards tasks, participants were explicitly informed about the probabilities, whereas in the Risky Gains task, probabilities were unspecified. Overall, the effects of emotional facial expressions on risky choice appear quite consistent and robust across task characteristics. As such, our results suggest that facial expressions can influence risk attitudes (risk seeking/risk aversion) and cannot be easily explained by changes in rationality, optimality, impulsivity, or depth of processing. Still, it would be interesting in future research to include a task in which the risky option has a lower expected value than the safe option, as this would even more stringently test the possibility that positive faces can increase risk seeking whereas negative faces increase risk aversion.

Before discussing implications of these results, it is important to mention some statistical limitations. First, the studies involving the 3-card paradigm (one part of Study 3, and Study 4) were underpowered: according to G-Power each study had about 50% power to detect the observed effect sizes, whereas SPSS 28 estimates for specific analyses were around 60–70%. However, the robustness of pattern across Study 3 and Study 4 gives us some confidence in the results. Note also that, when combined, the studies had an observed power of 84% (G-Power). Still, caution in interpretation of the results from this task is warranted. Second, *post-hoc* tests between individual expression conditions were two-tailed, but conducted without correction for multiple comparisons. Thus, while the overall valence effect appeared robust across paradigms, any differences between effects of specific facial expressions should be regarded with caution.

### Implications for emotion and decision

The current work extends previous work on the role of affect and emotion in judgment and decision-making. Earlier studies demonstrated that implicitly processed facial expressions influence simple judgments and behaviors (Niedenthal, 1990; Murphy and Zajonc, 1993; Marsh et al., 2005; Winkielman et al., 2005a; Anderson et al., 2012; Winkielman and Gogolushko, 2018; Yang and Yeh, 2018). Here, we show that emotional expressions can influence decisions in more complex tasks, such as choices between a certain option and a gamble, or among various gambles. As mentioned in the introduction, classic studies showed that neurological damage to mechanisms underlying affective feedback can either impair or improve decisions, depending on the structure of the decision environment (Bechara et al., 1997; Shiv et al., 2005). Later studies showed that facial expressions bias what participants assume and how they learn about the reward structure of the environment (Averbeck and Duchaine, 2009; Pittig et al., 2014). Here, we show that in healthy participants, implicit emotional cues exert valence-specific effects on risky decisions in a probabilistic setting.

The current research also differs from earlier studies examining how enduring moods (Isen et al., 1988) or qualitatively specific emotional feelings influence decisions (Johnson and Tversky, 1983; Raghunathan and Pham, 1999; Lerner and Keltner, 2001; Fessler et al., 2004). Such studies suggest that inducing feelings can influence choices by activating different cognitive appraisals (Lerner and Keltner, 2001), inferential mechanisms such as the “how-do-I-feel-about-it” heuristic (Clore and Huntsinger, 2007), concerns about changes in the current state (Isen et al., 1988), attempts at regulation (Leith and Baumeister, 1996), and a host of other mechanisms (for review, see Cohen et al., 2018). Accordingly, such studies often report unique effects of specific feelings, such as anxiety, anger, sadness, or guilt. In contrast, the current research focused on the influence of basic emotional stimuli, in the form of facial expressions presented as task-irrelevant cues, on a series of rapid, simple, and repeated choices. As argued by several theorists, implicit processing of such cues initially activates general positive and negative affective reactions, accounting for a broad positive–negative impact on immediate behavior and physiology observed in current studies (Lang, 1995; Cacioppo and Berntson, 1999; Russell, 2003; Barrett, 2017). Of course, with more processing resources (e.g., attention and time devoted to the stimulus, one’s own reaction, and the choice itself), the effects of facial expressions might be more differentiated. For example, if such facial expressions generated consciously experienced emotions of fear and anger, those emotions might differ in terms of associated appraisals (which cause, as well as result from, associated emotions). Those appraisals (e.g., certainty and control associated with anger, uncertainty, and helplessness associated with fear) might then generate opposing effects on risk attitudes (Lerner and Keltner, 2001).

What are the precise mechanisms by which implicit facial expressions influence risky decisions? We can only speculate about underlying processes, but suspect the influence to be implicit and direct, rather than mediated *via* inferences based on changes in subjective emotional experience. After all, in the current studies, as in previous research, the effects of facial expressions occurred under brief presentation conditions and were not mediated by explicitly reported changes in feelings (see also Winkielman et al., 2005b; Bornemann et al., 2012; Winkielman and Gogolushko, 2018). With that in mind, happy expressions could implicitly encourage exploratory behavior, perhaps by enhancing participants’ optimism, or their *implicit* expectation that a gamble will resolve positively (Slovic et al., 2002; Clore and Storbeck, 2006). Happy facial expressions could also transiently increase risk taking by highlighting the upside of the gamble (or downplaying its downside), *via* attentional or value-computation mechanisms (Paulus and Yu, 2012). At the neural level, this might involve transient activation (priming) of subcortical circuits in the nucleus accumbens, amygdala, and anterior insula, along with connections to higher cortical representations of value (e.g., Kuhnen and Knutson, 2005; Knutson et al., 2008; Phelps et al., 2014). The affective influence could also involve facial mimicry of emotional expressions because this bodily response facilitates processing of congruent valence concepts (Winkielman et al., 2018). This might be investigated using mimicry-blocking manipulations (Foroni and Semin, 2011; Davis et al., 2017). Future research should explore these and related questions, such as whether distinct affective, as opposed to purely cognitive, mechanisms push participants toward or away from risky options, and whether similar effects occur with non-facial emotional cues (*cf.* Furl et al., 2012).

In conclusion, the current studies demonstrate that common social stimuli such as emotional facial expressions can increase or decrease people’s risky choices, even when processed implicitly. Thus, these findings contribute to a growing recognition that judgment and decision-making requires input from affective as well as cognitive processes (e.g., Dukes et al., 2021).

## Data availability statement

The raw data supporting the conclusions of this article will be made available by the authors, without undue reservation.

## Ethics statement

The studies involving human participants were reviewed and approved by University of California San Diego IRB. The patients/participants provided their written informed consent to participate in this study.

## Author contributions

PW helped to design all studies, data analysis, and manuscript writing. JT helped to run the studies 1, 3, and 4, data analyses, and assisted with early drafts of the manuscript. BB helped to design, run, analyze, and write up Study 2, and helped with later drafts of the manuscript. BK and MP helped in design of all studies, data analysis, and manuscript writing. All authors contributed to the article and approved the submitted version.

## Funding

We appreciate support from NSF (BCS-0350687 to PW), NIMH (R01DA13186 and R01DA016663 to MP), NIDA (R03 020615 to BK), and Studienstiftung des deutschen Volkes (to BB).

## Conflict of interest

The authors declare that the research was conducted in the absence of any commercial or financial relationships that could be construed as a potential conflict of interest.

## Publisher’s note

All claims expressed in this article are solely those of the authors and do not necessarily represent those of their affiliated organizations, or those of the publisher, the editors and the reviewers. Any product that may be evaluated in this article, or claim that may be made by its manufacturer, is not guaranteed or endorsed by the publisher.
